# Role of RNA in Molecular Diagnosis of MADD Patients

**DOI:** 10.3390/biomedicines9050507

**Published:** 2021-05-04

**Authors:** Célia Nogueira, Lisbeth Silva, Ana Marcão, Carmen Sousa, Helena Fonseca, Hugo Rocha, Teresa Campos, Elisa Leão Teles, Esmeralda Rodrigues, Patrícia Janeiro, Ana Gaspar, Laura Vilarinho

**Affiliations:** 1 Research & Development Unit, Human Genetics Department, National Institute of Health Doutor Ricardo Jorge, 4000-055 Porto, Portugal; celia.nogueira@insa.min-saude.pt (C.N.); lisbeth.silva@insa.min-saude.pt (L.S.); 2 Newborn Screening, Metabolism & Genetics Unit, Human Genetics Department, National Institute of Health Doutor Ricardo Jorge, 4000-055 Porto, Portugal; ana.marcao@insa.min-saude.pt (A.M.); carmen.sousa@insa.min-saude.pt (C.S.); helena.fonseca@insa.min-saude.pt (H.F.); hugo.rocha@insa.min-saude.pt (H.R.); 3 Inherited Metabolic Diseases Reference Centre, São João Hospital University Centre, EPE, 4200-319 Porto, Portugal; teresaalmeidacampos@gmail.com (T.C.); e.leaoteles@gmail.com (E.L.T.); esmeralda.rodrigues@hotmail.com (E.R.); 4 Inherited Metabolic Diseases Reference Centre, Lisboa Norte University Centre, EPE, 1649-028 Lisboa, Portugal; patricia.janeiro@gmail.com (P.J.); ana.m.gaspar@chln.min-saude.pt (A.G.)

**Keywords:** glutaric aciduria type II, MADD, β-oxidation, *ETFDH*, NBS, RNA

## Abstract

The electron-transfer flavoprotein dehydrogenase gene (*ETFDH*) encodes the ETF-ubiquinone oxidoreductase (ETF-QO) and has been reported to be the major cause of multiple acyl-CoA dehydrogenase deficiency (MADD). In this study, we present the clinical and molecular diagnostic challenges, at the DNA and RNA levels, involved in establishing the genotype of four MADD patients with novel *ETFDH* variants: a missense variant, two deep intronic variants and a gross deletion. RNA sequencing allowed the identification of the second causative allele in all studied patients. Simultaneous DNA and RNA investigation can increase the number of MADD patients that can be confirmed following the suggestive data results of an expanded newborn screening program. In clinical practice, accurate identification of pathogenic mutations is fundamental, particularly with regard to diagnostic, prognostic, therapeutic and ethical issues. Our study highlights the importance of RNA studies for a definitive molecular diagnosis of MADD patients, expands the background of *ETFDH* mutations and will be important in providing an accurate genetic counseling and a prenatal diagnosis for the affected families.

## 1. Introduction

Multiple acyl-CoA dehydrogenase deficiency (MADD) is a rare autosomal recessive disorder of fatty acid, amino acid and choline metabolism that is also known as glutaric aciduria type II (MIM #231680) [1].

This disease primarily results from the absence and/or inactivity of either electron-transfer flavoprotein (ETF, encoded by alpha ETF (*ETFA*) and beta ETF (*ETFB*) gene) or electron-transfer flavoprotein ubiquinone oxidoreductase (ETF-QO, encoded by ETF dehydrogenase (*ETFDH*) gene) [2,3]. ETF and ETF-QO are functionally associated; they both conduct electron transfer from flavin adenine dinucleotide (FAD)-containing acyl-CoA dehydrogenases for fatty acid β-oxidation to the ubiquinone pool for mitochondrial respiration [4,5]. ETF-QO couples electron transfer between acyl-CoA (as the electron donor) and ubiquinone (as the electron acceptor).

This is a clinically heterogeneous disease in terms of age of onset and severity, which has been divided into three clinical forms: a neonatal-onset form with congenital anomalies (type I), a neonatal-onset form without congenital anomalies (type II), and a late-onset form (type III) [6,7]. The condition is clinically heterogeneous, ranging from a severe, neonatal form, presenting with hypoketotic hypoglycemia, metabolic acidosis, cardiomyopathy, and hepatomegaly, to a later-onset form characterized by proximal myopathy [8], Types I and II are severe, typically fatal, and characterized by nonketotic hypoglycemia, metabolic acidosis, and accumulation and excretion of metabolites, while Type III is milder, more variable, and characterized by recurrent episodes of hypoglycemia, metabolic acidosis, vomiting, and muscle weakness during catabolic stress [9,10,11].

Patients are identified through clinical presentation and, in some countries, such as Portugal, also via an expanded newborn screening (NBS) program [12,13]. The biochemical profile of MADD patients usually reveals increased levels of aliphatic mono and dicarboxylic acids as well as acylglycine conjugates in urinary organic acids, and increased levels of long and medium chain acylcarnitines in the blood [14].

Treatment options include dietary fat and protein restrictions, fasting avoidance, and supplementation with carnitine, glycine, and riboflavin. Riboflavin supplementation is also known to strikingly improve clinical symptoms and metabolic profiles in some MADD patients, particularly those with late-onset forms [2,15]. Despite early identification and treatment, neonatal mortality remains high [16,17].

More than one hundred mutations have been described so far in the *ETFA*, *ETFB* and *ETFDH* genes. However, *ETFDH* gene mutations have been reported to be the major cause of MADD with 236 published until now, of which only one deep intronic and three gross deletions were reported [18,19,20]. This gene is mapped to 4q33-q35 and consists of 617 amino acid residues [21].

The clinical phenotype depends on the location and nature of these mutations. Null mutations severely affecting mRNA expression, processing and/or stability are associated with lethal forms of the disease, while missense mutations that afford residual enzyme activity are associated with milder clinical outcomes [6]. Nevertheless, as in many other metabolic disorders, clear genotype–phenotype correlations remain elusive, especially within the milder forms of the disease, in which it is expected that the variant proteins are partially functional [21].

Here, we report four patients of early onset MADD, detected by NBS, the method that is usually used in the molecular characterization of NBS patients, in which only a heterozygous mutation was detected by DNA sequencing. However, in this present work, we had to perform RNA studies in order to identify the second causative mutation of these patients, despite the few mutations published at this level.

## 2. Patients and Methods

### 2.1. Patients

**Figure 1:**
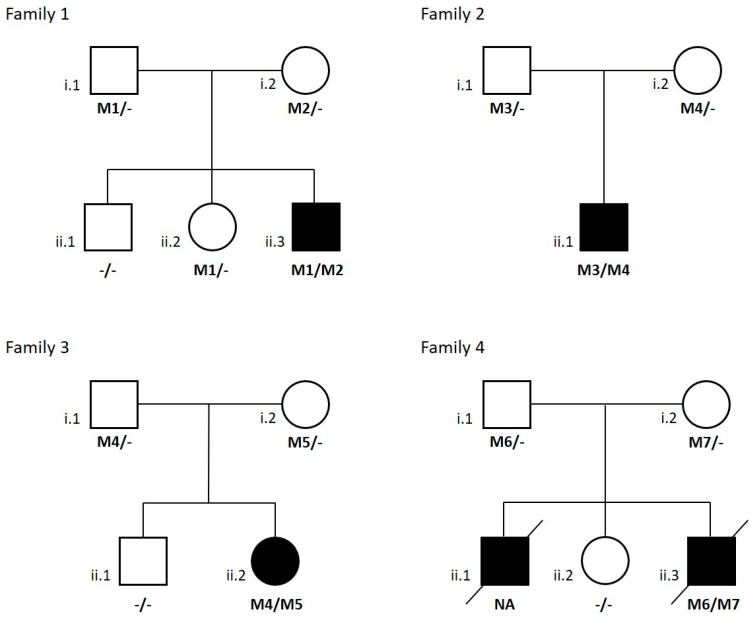
Families’ pedigrees and genotype data from four MADD families. Symbols: square, male; circle, female; filled, affected individual; diagonal line, deceased; NA, not available. Mutations found in *ETFDH* gene are shown below each symbol: -, wild-type allele; M1, p.Val324Met (c.970A>G); M2, c.35-768A>G; M3, p.Arg41* (c.121C>T); M4, c.35-1008 T>G; M5, p.Leu550Valfs*4 (c.1648_1649delCT); M6, c.34_607del; M7, p.Arg155Gly (c.463A>G).

Using NBS, we found 10 MADD patients since 2004, and all were genetically characterized by the conventional method, DNA sequencing, except for four patients (P1–P4). They are from four unrelated families (Figure 1) recruited from medical centers located in Portugal that were enrolled in the current study. Informed consent for participation in this study was obtained from the parents of all investigated subjects in agreement with the Declaration of Helsinki and approved by the ethical committees of the centers participating in this study, where biological samples were obtained.

### 2.2. Methods

#### 2.2.1. Samples

Genomic DNA was extracted from individuals’ peripheral blood using Bio Robot EZ1 (Qiagen, Hilden, Germany), according to standard procedures. Total RNA was extracted from PAXgene Blood RNA tubes (PreAnalytiX, QIAGEN, Germantown, MD, USA) using the PAXgene Blood RNA kit (Preanalytix, QIAGEN, Germantown, MD, USA), according to the manufacturer’s recommendations. Control RNA was extracted from individuals who did not carry *ETFDH* mutations. The DNA and RNA were quantified with the Nanodrop 2000 C spectrophotometer (Thermo Fisher Scientific, Waltham, MA, USA).

#### 2.2.2. DNA Sequencing

For DNA studies, we used the technology of Next Generation Sequencing (NGS) that was performed in a MiSeq Illumina platform using a SureSelect QXT kit (Agilent Technologies, Santa Clara, CA, USA) to captured three genes of interest (*ETFA*, *ETFB* and *ETFDH*). Variant calling and annotation were performed using available commercial programs, such as Surecall (Agilent Technologies, Santa Clara, CA, USA) and wAnnovar (wannovar.wglab.org/, accessed on 1 March 2021). Variants were filtered taking into account: (i) the type of mutation (missense, frame-shift, stop-gain or stop-loss, and splice-site variants), (ii) in silico predictors (SIFT, PolyPhen-2, MutationTaster, accessed on 1 March 2021) [22,23,24] and presence in databases (dbSNP, 1000 Genomes, HGMD professional, ClinVar, ExAC, OMIM and gnomAD), and (iii) the population frequency (variants with a minor allele frequency (MAF) >1% in the 1000 Genomes Project (http://www.1000genomes.org, accessed on 1 March 2021) and Exome Variant Server databases (http://evs.gs.washington.edu, accessed on 1 March 2021) were filtered out).

#### 2.2.3. RNA Sequencing

The cDNA was generated using the SuperScript III First-Strand Synthesis SuperMix for RT-PCR (Invitrogen, Thermo Fisher, Waltham, MA, USA) following the manufacturer’s instructions. *ETFDH* cDNA was amplified in a 25 µL reaction containing: 12.5 μL of 1X ImmoMix™ Red (Bioline, London, UK), 1.5 to 2.5 μL of cDNA and 0.4 μM of each forward/reverse specific primer (Table 1). PCR cycling conditions were as follows: initial denaturation at 95 °C for 10 min; 35 cycles of 95 °C for 30 s, 45 s at annealing temperature, extension at 72 °C for 60 s and a final extension for 10 min at 72 °C. The PCR amplicons were purified and Sanger sequencing was performed. To identify the variants that caused alternative splicing, we designed specific primers to sequence *ETFDH* cDNA and a region of interest in intron 1 of this gene (Table 1). In silico analysis of the identified deep intronic variants was conducted by running two independent algorithms for splice signal detection: NetGene2 (http://www.cbs.dtu.dk/services/NetGene2/, accessed on 1 March 2021), Alamut Visual v.2.11 (http://www.interactive-biosoftware.com/alamut-visual/, accessed on 1 March 2021) and Human Splicing Finder (http://www.umd.be/HSF/, accessed on 1 March 2021).

#### 2.2.4. Confirmatory Sanger Sequencing Analysis

All variants detected that had the potential to be disease-causing were confirmed by Sanger sequencing using a BigDye Terminator Cycle Sequencing Kit (Applied Biosystems, Foster City, CA, USA), and analyzed on an ABI PRISM 3130xl Genetic Analyser (Applied Biosystems, Foster City, CA, USA). Co-segregation studies were also performed in DNA or RNA from additional family members.

## 3. Patients Presentation

### 3.1. Patient 1

**Figure 2:**
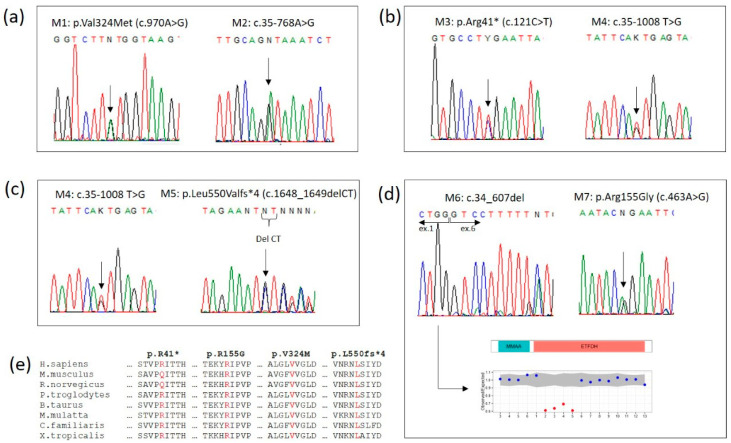
Electropherograms of *ETFDH* mutations from (**a**) family 1, (**b**) family 2, (**c**) family 3 and (**d**) family 4, showing the deletion detected by Copy Number Variation analysis. (**e**) Sequence alignment among 8 vertebrates around the sites of the four exonic mutations. The four identified mutations are conserved across species.

A 3-day-old boy was born at full-term to nonconsanguineous parents after an uneventful pregnancy. Physical examination revealed a weight of 3.500 g. No dysmorphic features were noted. At 36 h of life, the neonate presented hypotonia, hypoglicemia and a seizure episode. Biochemical study revealed elevation of ALT (148 U/L) AST (212 U/L) and CK (1868 U). Tranfontanellar, abdominal and cardiac US were unremarkable. At 3 days of age, the newborn screening sample showed a combined elevation of long and medium chain acylcarnitines (Table 2), supporting a diagnosis of MADD, starting riboflavin, carnitine and Q10, with low protein and low-fat metabolic formula. Due to feeding difficulties and poor weight gain, a gastrostomy tube was placed at 10 months of age. At 20 months, the patient was admitted to the hospital due to an episode of fever and vomiting. Now, at 3 years of age, he exhibits normal growth and development. Mutational analysis of the *ETFDH* gene revealed two novel disease-causing variants: p.Val324Met (c.970A>G) and c.35-768A>G (Figure 2a).

### 3.2. Patient 2

An 11-day-old boy was born at full-term to nonconsanguineous parents after an uneventful pregnancy. He was in good health and feeding well at that time. A newborn screening obtained at that time revealed elevations of multiple acylcarnitines in a pattern consistent with a biochemical diagnosis of MADD (Table 2), and, at that time, he was admitted to the hospital for investigation, without any symptoms, poor feeding or vomiting. The analytical evaluation showed increases in cardiac biomarkers and lactate. An abdominal ultrasound showed a mild hepatomegaly and the electrocardiogram was normal. Mutational analysis subsequently revealed that the patient was heterozygous for two mutations in the *ETFDH* gene: p.Arg41* (c.121C>T), already described in the literature, and a novel deep intronic variant, c.35-1008T>G (Figure 2b). He initialized treatment with riboflavin, and had normal growth and developmental milestones.

### 3.3. Patient 3

An 11-day-old girl was born at full-term to nonconsanguineous parents after an uneventful pregnancy. A brief episode of hypoglycemia occurred at the first day of age. The newborn screening was reported as positive for MADD, with elevations of multiple acylcarnitines (Table 2), at the 11th day of life. Biochemical evaluation (ALT; AST; CK), cardiac and abdominal US were normal. Treatment was started with low protein, low fat metabolic formula, riboflavin supplementation, carnitine and Q10. Mutational analysis revealed an already described mutation in the *ETFDH* gene, p.Leu550Valfs*4 (c.1648_1649delCT), and a novel deep intronic variant, c.35-1008T>G (Figure 2c). She had exhibited normal growth and development up until the time of the study.

### 3.4. Patient 4

A 3-day-old boy was born at term after a normal pregnancy with a weight of 2780 g. He was the third child of nonconsanguineous parents. The first child of this couple died from sudden death at 36 h, presented in the autopsy hepatic steatosis, which was suggestive of metabolic disease. At the second day of life, patient 4 was admitted to the hospital Emergency Department due to several episodes of hypoglycemia. A newborn screening sample, obtained at 3 days of age, was reported as positive for MADD, with elevations of multiple acylcarnitines profile (Table 2). A low-protein, low-fat metabolic formula was started with riboflavin supplementation. To further confirm the diagnosis, we identified a heterozygous compound in the *ETFDH* gene with an already described missense mutation and a novel gross deletion: p.Arg155Gly (c.463A>G) and c.34_607del, respectively (Figure 2d). The placement of a gastrostomy tube at 4 months was performed to attempt to reduce the risk of metabolic decompensation. At 24 months of age, he presented to the Emergency Department due to an enteric intolerance, vomiting, metabolic acidosis and tachypnea. The child experienced a cardiorespiratory arrest. During resuscitative efforts, ventricular arrhythmias were observed but could not be converted to a normal rhythm. Resuscitation was unsuccessful and the child passed away.

## 4. Discussion

We focus our study on 4/10 unrelated MADD patients, detected through expanded NBS, whose incidence in our country is 1:142,204 newborns. DNA sequencing was used to confirm all patients except four, for whom an RNA strategy was required to identify the second causative mutation. These four patients were classified as type II without congenital anomalies. Molecular diagnosis revealed seven mutations in *ETFDH* gene, with four of them being novel variants. The molecular confirmation of MADD diagnosis in the studied patients was just possible due to the RNA studies carried out, as three of these novel variants affect the splicing process. The novel variants identified were a missense variant (p.Val324Met (c.970A>G)), a gross deletion (c.34_607del) affecting exons 2 to 5, and two deep intronic variants (c.35-768A>G and c.35-1008T>G) that induce alternative splicing and generate aberrant transcripts [20,25]. In this study, we also identified mutations already described in the literature, such as p.Leu550Valfs*4 (c.1648_1649delCT) [13], p.Arg41* (c.121C>T) [1] and p.Arg155Gly (c.463A>G) [21].

**Figure 3:**
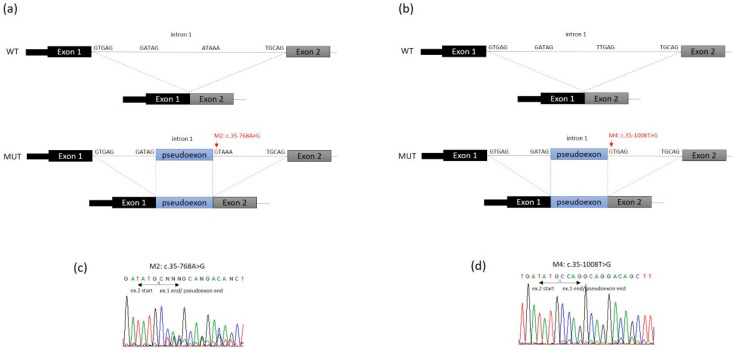
Schematic representation of *ETFDH* region including exon 1, intron 1 and exon 2. WT: structure of wild-type *ETFDH* transcript (exons 1–2). MUT: structure of *ETFDH* transcript generated due to (**a**) c.35-768A>G mutation (red, arrow) and (**b**) c.35-1008T>G mutation (red, arrow). Intron splice site acceptor and donor sequences are shown. In the two cases (**a**,**b**) the pseudoexons (blue) result from activation of the 5′ donor site that also results in preferential use of the existing 3′ acceptor site (GATAG) at the 5′ end of the pseudoexon to effect splicing. (**c**,**d**) Electropherograms showing the cDNA sequences of patients with the heterozygous mutations c.35-768A>G and c.35-1008T>G, respectively.

In patient 1, two novel disease-causing variants were identified: a missense variant considered likely to be pathogenic through bioinformatic analysis, which results in the change of a conserved position through different homologues (p.Val324Met (c.970A>G)) (Figure 2e), and a deep intronic variant (c.35-768A>G), which creates a splice donor site used for the insertion of a new pseudoexon between exons 1 and 2 (Figure 3a). The above loci were inherited from the parents, each of whom carried only one heterozygous variant. Molecular diagnosis of his mother showed the deep intronic heterozygous variant c.35-768A>G, his father and sister showed the missense heterozygous variant p.Val324Met (c.970A>G), and his brother did not show any of these mutations (Figure 1).

In patient 2, the mutational analysis revealed an already described mutation in the *ETFDH* gene, p.Arg41* (c.121C>T), and a novel deep intronic variant c.35-1008T>G, which create a splice site/pseudoexon between exons 1 and 2 (Figure 3b). Segregation studies in the parents showed the heterozygous nonsense mutation in the father and the deep intronic variant in the mother (Figure 1).

In patient 3, a heterozygous compound was identified with an already-described frameshift mutation (p.Leu550Valfs*4 (c.1648_1649delCT)) and a deep intronic variant (c.35-1008T>G), which affect the splicing process, causing the insertion of a pseudoexon between exons 1 and 2 (Figure 3b). Both parents confirm the segregation of these mutations and her brother did not present any of them (Figure 1).

In patient 4, a missense mutation already described by our group (p.Arg155Gly (c.463A>G)) was identified in this patient with heterozygosity, as well as a novel gross deletion which revealed a heterozygous loss of approximately 5 kb. Initially, this patient appeared to be homozygous for the identified missense mutation, however sequence analysis of parental DNA demonstrated that his father did not carry this mutation and that the mother was a carrier. cDNA sequencing of exons 1 to 6 of *ETFDH* showed the presence of the heterozygous deletion, which includes exons 2–5, in the proband and in his father (Figure 1).

In clinical practice, accurate identification of pathogenic mutations is fundamental, particularly with regard to diagnostic, prognostic, therapeutic and ethical issues. Approximately 15–50% of all human disease mutations have been shown to affect the work of the classic splicing pathway [26]. The list of splicing mutations described in the literature has grown enormously, and is expected to increase even further as RNA-sequencing (RNA-seq) abilities improve [25,27,28,29]. Therefore, we are implementing this technology in our lab, using not only RNA extracted from blood, but also from skin fibroblasts or muscle, since it will allow a more precise molecular diagnosis, making an impact on the “missing mutations”.

## 5. Conclusions

In conclusion, this study expands the clinical and genetic spectrums of MADD presentations detected by NBS, highlighting the importance of RNA sequencing for a definitive molecular diagnosis of this disease. Transcriptome analysis using RNA-seq technology, which is currently being implemented in our lab, will be essential to provide evidence of a second variant to complement an already-identified heterozygous mutation. Early diagnosis and timely treatment play an important role in reducing the risks of morbidity and mortality associated with MADD, allowing an accurate genetic counseling and a prenatal diagnosis for the affected families.

## Figures and Tables

**Table 1:** Primers to sequence *ETFDH* cDNA and a region of interest in intron 1.

Primers cDNA	Sequence	Annealing Temperature
F1	TGTTGTGTCCGACCGAGA	60 °C
R1	TGGCTCCGTATGCAATCC	
F2	AACGCCGTGAAGCAAGAG	60 °C
R2	CCACTTTTCATTGCTGTGTGA	
F3	TCCTAGCATTCGGCCAAC	60 °C
R3	CCCGTAATTTCTTTATGGGACA	
**Primers Intron1**	**Sequence**	**Annealing Temperature**
i1F1	TTCTCCCTAATTTGAAATGGTAT	60 °C
i1R1	GGCAGGTACCCTAGCATCAA	
i1F2	CTGCCAAGGAGTTGAGAAAA	60 °C
i1R2	GCAATCTCAGCTCACCACAA	

**Table 2:** Acylcarnitines profile of four MADD patients detected through newborn screening.

Metabolite Marker	Metabolite Concentrations (μM)				
	Reference Values	Patient 1	Patient 2	Patient 3	Patient 4
Free carnitine (C0)	>9.13	35.98	43.14	36.20	21.91
Glutarylcarnitine (C5DC)	<0.20	0.39	0.17	0.10	1.75
Butyrylcarnitine (C4)	<0.97	1.10	1.16	0.90	3.11
Hexanoylcarnitine (C6)	<0.20	1.96	0.48	0.90	1.28
Octanoylcarnitine (C8)	<0.30	3.96	1.13	2.51	6.40
Decanoylcarnitine (C10)	<0.44	4.31	1.75	3.73	2.96
Dodecanoylcarnitine (C12)	<0.51	3.29	2.32	4.36	2.23
Dodecenoylcarnitine (C12:1)	<0.46	1.14	0.64	0.88	0.62
Tetradecanoylcarnitine (C14)	<0.59	3.52	2.48	2.71	3.12
Tetradecenoylcarnitine (C14:1)	<0.46	2.98	2.47	3.46	1.78

## Data Availability

Generated during the study.

## Abbreviations

MADD	Multiple acyl-CoA dehydrogenase deficiency
NBS	Newborn screening
ETF	Electron-transfer flavoprotein
FAD	Flavin adenine dinucleotide
NGS	Next Generation Sequencing
